# Influence of Anti-Infective Periodontal Therapy on Subgingival Microbiota Evaluated by Chair-Side Test Compared to qPCR—A Clinical Follow-Up Study

**DOI:** 10.3390/antibiotics11050577

**Published:** 2022-04-26

**Authors:** Oliver Laugisch, Thorsten M. Auschill, Anne Tumbrink, Anton Sculean, Nicole B. Arweiler

**Affiliations:** 1 Department of Periodontology and Peri-Implant Diseases, Philipps-University, 35039 Marburg, Germany; oliver.laugisch@uni-marburg.de (O.L.); auschill@med.uni-marburg.de (T.M.A.); anne.tumbrink@gmx.de (A.T.); 2 Private Practice, 48324 Sendenhorst, Germany; 3 Department of Periodontology, School of Dental Medicine, University of Bern, 3010 Bern, Switzerland; anton.sculean@zmk.unibe.ch

**Keywords:** periodontal therapy, supra- and subgingival debridement, anti-infective therapy, adjunctive antimicrobials, antibiotics, diagnosis, effectiveness, chair-side, point of care, evaluation

## Abstract

A chair-side test (CST) for five periodontal pathogens (*Aggregatibacter actinomycetemcomitans*, *A.a.*; *Porphyromonas gingivalis*, *P.g.*; *Prevotella intermedia*, *P.i.*; *Treponema denticola*, *T.d.*; *Tannerella forsythia*, *T.f.*) was compared with qPCR in a previous clinical study on 100 periodontitis patients at first diagnosis (T0). Following non-surgical treatment alone (SRP) or in combination with systemic or local antibiotics, 74 patients (57.4 ± 13.5 years) were again tested at the same sites from 14 to 24 months after T0. Bacterial elimination (%; compared to T0) was determined for each single species and compared between both test systems. In all patients, all five pathogens could not be fully eliminated regardless of therapy or test method. Tested with CST, the mean elimination ranged from 90% for SRP + Amoxicillin/Metronidazole to 59.13% for SRP only. The corresponding qPCR values were 30% and 29.6%. Only *A.a.* was eradicated in 100% by SRP + Amoxicillin/Metronidazole tested by CST, and it was 80% when qPCR was the test method. CST agreed with qPCR in 98.7% in the detection of *A.a.*, and 74.3%, 78.4%, 73.0%, and 48.7% for *P.g.*, *P.i.*, *T.d.*, and *T.f.*, respectively. Neither conventional treatment nor the additional use of antibiotics—even with the correct indication—could completely eradicate the tested pathogens or prevent pocket reinfection.

## 1. Introduction

Periodontal pathogens are the main cause of periodontal disease [1]. These bacteria should be removed both supra- and subgingivally (in a first and a second treatment phase). Subgingival debridement, also called scaling and root planing (SRP), using manual or sonic/ultrasonic devices is strongly recommended in a recent S3-treatment guideline [2]. Since anti-infective therapy aims to reduce subgingival microbiota and inflammation, mechanical instrumentation can be supported by systemic antibiotics at special indications [2]. By triggering a cascade of immune reactions, periodontal pathogens are a challenge not only for oral but also for general health [3]. This underlines the importance of investigating the bacterial spectrum of periodontal pockets. Within the plethora of bacteria, the focus was and still is on five pathogens with a strong association to disease initiation and progression: *Aggregatibacter actinomycetemcomitans*, *Porphyromonas gingivalis*, *Tannerella forsythia*, *Treponema denticola*, and *Prevotella intermedia* [4]. 

The need for microbiological diagnostics and bacterial testing prior to periodontal therapy is controversially discussed [2,5,6,7,8,9,10,11]. In the context of the currently emerging field of personalized medicine, microbiological tests can be a tool to facilitate a targeted therapy including antibiotics to eliminate periodontal pathogens based on the subgingival microbiologic profile. Despite all of the pros and cons, it can be summarized that microbiological tests are an additional diagnostic tool similar to those in personalized general medicine, e.g., measuring blood pressure or blood sugar, helping to interpret hypertension or diabetes and optimize the following therapy [12]. 

So far, the literature could not show if previous microbiological testing leads to more antibiotic prescription or to a more responsible and lesser use of antibiotics [9,11,13]. 

The recent S3-treatment guideline [2] proposed—supported by a majority of underlying research—the adjunctive use of systemic antibiotics in specific patient categories (e.g., generalized stage III periodontitis in young adults) and the combination of Amoxicillin and Metronidazole as having the most pronounced effect on clinical outcomes. However, when it comes to administration in specific cases, studies show high variations in the indications and the selection of antibiotics or their combinations, doses and administration times [14]. Often they are prescribed on an empirical basis [15] or even without indication [9]. 

Long-term studies investigating the microbiological profile after periodontal therapy are rare and data after non-surgical periodontal therapy including antibiotics are in demand [13], as only data on clinical parameters are available [16]. 

Since many pathogens present in the periodontal pockets are not cultivable [17,18], standard microbiological cultivation procedures cannot be applied. Instead, molecular biologic procedures, especially the use of a quantitative polymerase chain reaction (qPCR) to determine bacterial DNA, are the golden standard and have been used in dentistry for decades [18,19,20,21]. These are of high sensitivity and specificity and do not require living bacterial cultures, but are also time consuming, as samples need to be sent to an external laboratory [5].

For years, chair-side tests (CSTs) targeting bacteria, viruses, or other biomarkers have been relegated to a niche existence. Due to the COVID-19 pandemic, they recently experienced a strong upswing due to their advantages of fast and on-site testing. A newly developed RNAase-based CST enables the determination of the five most relevant periodontal pathogens on the basis of microarrays in a time span of about 20 min. Recently, its sensitivity and specificity was evaluated in 100 periodontitis patients and 25 periodontally healthy study participants [5]. In comparison to quantitative real time PCR (qPCR; DNA-based), as the gold-standard in microbiological diagnostics, the CST had a good correlation and a somewhat lower sensitivity towards a qPCR [5]. As a follow up, all periodontitis patients (100) of this former study received systematic anti-infective periodontal treatment comprising subgingival scaling and root planing (SRP) alone or in combination with different systemic or local antibiotics according to their microbiologic profile. 

It was the aim of this follow-up study to evaluate the microbiological response 14 to 24 months after anti-infective therapy and compare the results of the CST with a qPCR again. 

## 2. Results

### 2.1. Patients and Periodontal Therapy

A total of 74 out of the previous 100 patients with an age of 57.4 ± 13.5 years (33 females, 45%; 41 males, 55%) could be included in this clinical study for a microbiologic follow-up 14 to 24 months (T1) after first diagnosis (T0). A total of 26 patients declined to participate. According to previous clinical diagnosis and microbiological test results all patients had received a systematic periodontal therapy with scaling and root planing (SRP). Whereas 44 were treated with SRP alone, 30 patients received adjunctive antibiotics according to the microbiologic spectrum, as shown in detail under Material and Methods. All patients were given a re-evaluation 3–4 months after active therapy and were invited to a maintenance program (supportive periodontal therapy, SPT). 

Out of these 74 patients, 49 patients (66.2%) kept SPT at the Department of Periodontology & Peri-Implant Diseases at the Dental School in Marburg. Within this cohort, adherence with SPT was better if patients were initially treated with additional antibiotics (73.3%) than with SRP alone (61.4%). Non-adherence with SPT was more frequent in males (*n* = 17; 68.0%) than in females (*n* = 8; 32.0%).

### 2.2. Bacterial Elimination (in %) Depending on Treatment Modalities 

The rate of samples with at least one positive pathogen detection after treatment (T1) in relation to initial diagnosis (T0) sorted by therapy is shown in Table 1. In none of the patients, all five periodontal pathogens could be fully eliminated regardless of therapy or test method. Tested with CST, the mean elimination ranged from 90% for SRP + Amoxicillin and Metronidazole and 59.1% for SRP only. The corresponding qPRC values were 30% and 29.6%. 

### 2.3. Elimination Rate of Single Species 

Table 2 represents the elimination rate (in %) for each single species again grouped by different therapy options as well as the agreement of both test systems. *A.a.* was eradicated in 100% by SRP + Amoxicillin/Metronidazole tested by CST and 80% when qPCR was the test method. The lowest elimination rates were found for *P.g.* (40%) by CST when Azithromycin was administered and for *P.i.* by qPCR when SRP only was the therapy (22.2%) (Table 2).

**Figure 1:**
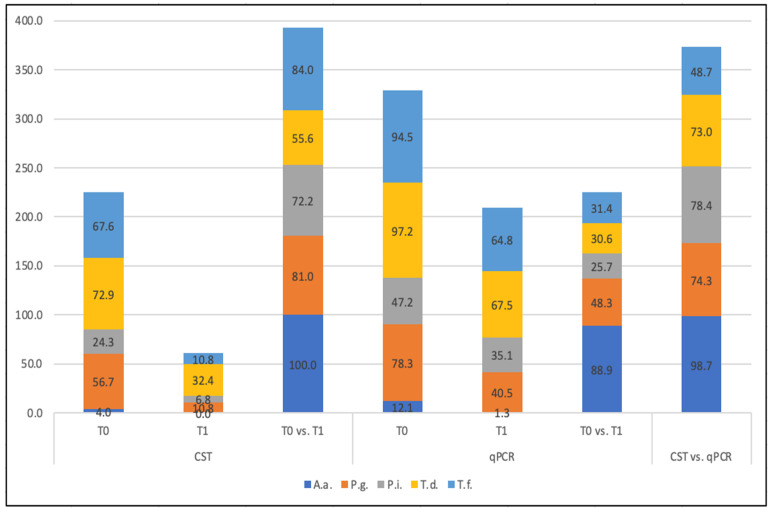
Samples (%) tested positive for single species (*A.a.*: *Aggregatibacter actinomycetemcomitans*, *P.g.*: *Porphyromonas gingivalis*, *T.f.: Tannerella forsythia*, *T.d.*: *Treponema denticola*, and *P.i.*: *Prevotella intermedia)* at T0 and T1 tested by CST and qPCR, their bacterial elimination rate in % (T0 vs. T1), and % agreement of both tests.

Irrespective of the treatment modalities, 100% *A.a.*, 81% *P.g.*, 72.2% *P.i.*, 55.6% *T.d.*, and 84% *T.f.* elimination rates could be determined by CST. The corresponding qPRC-values were 88.9% for *A.a.*, 48.3% for *P.g.*, 25.7% for *P.i.*, and 30.6% as well as 31.1% for *T.d.* and *T.f.* (Figure 1).

### 2.4. Comparison of CST with qPCR 

The agreement of both test systems depending on treatment and/or bacteria is plotted in Table 1 and Figure 1. For *A.a.*, CST conformed with qPCR in 98.7% of cases. *P. gingivalis* detection was in accordance of 74.3%. For *P. intermedia*, *T. denticola*, and *T. forsythia* tests, they agreed in 78.4%, 73.0%, and 48.7%, respectively, resulting in an average agreement of 74.6%. When grouped by treatment, an agreement of both tests has been shown between 40% and 90% (Table 1).

## 3. Discussion

This clinical study revealed that (1) all non-surgical periodontal therapy approaches were able to reduce five key pathogens (*A.a.*, *P.g.*, *P.i.*, *T.d.*, and *T.f.*) and (2) the CST presents a quick and useful alternative to a qPCR to check for successful bacterial elimination after therapy.

The study was designed as a follow up of a recently published study [5], where the sensitivity and specificity of this newly developed CST were already tested and compared with a qPCR as a reference method. CST reached a sensitivity of 87.8%, and qPCR of 94% (with regard to clinical diagnosis), and the correlation was classified as high. Specificity, tested on healthy participants, was 100% for both methods [5]. It was already pointed out that CST had a higher detection limit (10^4^) than qPCR (10^2^). This could be explained by the fact that the RNAase method used in CST detects living bacteria only. These can proliferate and could still cause inflammation in the periodontal pocket. In contrast, the reference method is DNA-based and could also detect dead bacteria or free DNA. In this context, Polonyi et al. [22] found that DNA is slowly degraded after vitality-loss. Under certain circumstances, it might even be detectable years after cell death. Thus, they recommend the use of RNA-based detection methods, especially to verify the successful elimination of periodontal pathogens. 

Meanwhile, within the scope of the COVID-19 pandemic, it is broadly known (even among non-scientists) that chair-side tests (or point-of-care tests) exhibit a lot of benefits. However, they are not as sensitive as qPCR when detecting SARS-CoV-2. In the case of this highly pathogenic virus, a verification with qPCR is surely obligatory. 

In view of the microbial profile of patients’ pockets, it has to be considered that qPCR detects also DNA fragments of dead bacteria, leading to a possible overestimation. 

With this in mind, Loozen et al. (2011) [21] modified qPCR by adding Propidium Monoazid (PMA) in order to amplify and to detect viable/ bacteria only. PMA penetrates non-viable bacteria and blocks PCR amplification by intercalcation in double-strain DNA. While PMA-qPCR and PCR determined a massive bacterial reduction after antibiotic-treatment, qPCR did not. A reason might be that bacterial loads, necessarily not being pathogenic, are highly amplified by qPCR. 

While for initial diagnostics before therapy no differences were observed [22], this can become more relevant for the re-evaluation of anti-infective therapy, including antibiotics. Thus, a CST could be sufficient to choose the most appropriate and personalized treatment option and, moreover, to evaluate the effectiveness of anti-infective treatment.

When comparing the elimination rates of both test systems after therapy, CST showed only half the rate of qPCR when SRP, SRP + Clindamycin, or SRP + local Doxycycline were the therapy options. In contrast, when SRP + Amoxicillin/Metronidazole or SRP + Azithromycin was administered, discrepancies were higher, ranging from only a third (SRP + A/M) to only one eighth (SRP + A). It can be assumed that these therapy options left over a lot of dead bacteria, which were detected by qPCR but not by CST. This is supported by the fact that these therapy options were especially chosen in patients with a high degree of inflammation and bacterial load. 

Most studies compare different microbiological tests at the point of initial diagnosis and not after therapy [16,18,19,22,23]. As only data on clinical parameters are available [16], the long-term effects of reducing or eradicating periodontal pathogens after non-surgical periodontal therapy including antibiotics are demanded [13]. This underlines the importance of our dataset.

It should, however, be considered that long-term follow-ups often do not show the success of one therapy, but rather the effectiveness of supportive periodontal therapy (SPT) and the level of personal oral hygiene, which are the most important factors to prevent bacterial re-infection [24,25]. Sokransky et al. [26] published a study with a comparable observation period. Although stable and clinically improved conditions had been seen over a period of 2 years, the number of pathogens had increased already after 6 months. Therefore, a therapeutic goal to prevent periodontal destruction is rather the control of periodontal pathogens on a low level than their total eradication [26].

Since adherence (formerly named compliance) to SPT was not a requirement or inclusion criterium for the follow-up in this study, one third did not participate in the SPT of the department, while two thirds (66%) received SPT in an interval of 3 to 6 months. This is a higher rate than generally reported in the literature. Fenol and Mathew [27] have shown that only 48.1% were compliant. It should, however, be kept in mind that not only the frequency, but also the quality of SPT plays an important role for the success of any treatment. An interesting finding of this present study is that patients with SRP and additional antimicrobials had a better compliance (73%) than those receiving SRP alone (61%). Obviously, the type of periodontal treatment seemed to be one of the key factors for adherence. In line with this finding, it has been shown that additional periodontal surgery is positively associated with adherence (formerly named compliance), concluding that the severity of periodontal disease and its destruction underlines the need for SPT, as patients are in fear of losing teeth [28]. Our dataset also showed that adherence was higher in females. This is in line with the literature showing females being more compliant [29,30], although it is mentioned that during long-term SPT, males yielded less bleeding on probing compared to smokers and females, respectively [31]. 

With CST, the poorest elimination rate of pathogens was shown with SRP alone (59%), followed by SRP + Clindamycin (67%), and SRP + local Doxycycline (67%); however, the latter two included less than 10 patients. The combination of Amoxicillin/Metronidazole and Azithromycin showed bacterial reductions of 90% and 72.8%, respectively, representing the therapy option with the highest elimination rates. In former studies, the administration of Azithromycin over a three-day period was a good alternative to Metronidazole in order to eliminate *P. gingivalis* due to its broad spectrum against aerobic and anaerobic Gram-negative microorganisms and its long half-life in periodontal tissue [32,33,34]. However, when taking a closer look at *P.g.* elimination, both test methods showed a reduction of around 40–45% only. Oteo [34] documented distinct clinical improvements and a significant reduction of *P. gingivalis* up to 6 months after SRP and Azithromycin in comparison with SRP and placebo. Similar to our study, SRP was carried out in two visits within a 7-day period, and the intake of Azithromycin started after finishing the second debridement. The 3- and 6-month results showed a tendency to an increase of *P. gingivalis*. This tendency could be an explanation for the somewhat lower elimination after our 14–24 months follow-up. 

So far, classical antibacterial agents and antibiotics are the recommended therapy options (S3 guideline) [2], but more and more alternative therapy options come into focus [35,36,37]. In different studies, probiotics, prebiotics, and other natural agents could show promising data to support non-surgical periodontal therapy; however, this has not yet resulted in a scientific recommendation (S3 guideline). 

In sum, the indicated therapy options, including mechanical debridement alone or with antibacterial agents as adjuvants, were able to eliminate the bacterial load in the pockets to a different extent. Both microbiological test systems revealed partly high discrepancies, which are, however, explained by differences in detecting viable and dead bacterial DNA. While the sample size of 74 patients still outnumbered the required sample size of 50, the groups comprising the different antibiotic therapies, especially for local antibiotics, were very small. Nevertheless, the data presented descriptively provide an interesting outlook for the microbiological effects of anti-infective periodontal therapy. 

## 4. Materials and Methods

### 4.1. Study Center and Subjects

The study was a follow-up of a former study [5] and was approved by the medical ethics committee of the Philipps University of Marburg (#29/13). 

**Figure 2:**
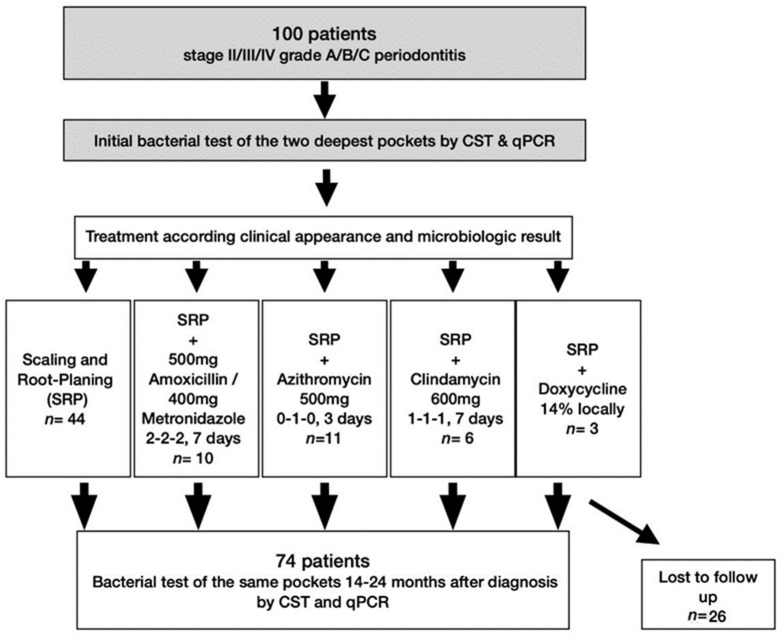
Flow chart of the study, the previous study parts are greyed out.

All 100 patients from the former study (excluding healthy participants) were invited 14–24 months (T1) after first microbiological testing (T0), meaning 10–12 months after an anti-infective periodontal treatment. A total of 74 of 100 initially treated patients could be included in this follow-up analysis—26 were lost due to relocation or were unwilling to participate in an additional study (Figure 2). All participants received the same three-digit identification number of the previous study for pseudonymization and signed (again) an informed consent prior to participation.

### 4.2. Study Design and Procedure

This clinical study was structured in accordance with the guidelines from CONSORT (http://consort-statement.org/, accessed on 16 March 2022) and in full accordance with ethical principles, including the World Medical Association Declaration of Helsinki (version 2008) and the current guidelines of the United States’ Food and Drug Administration (FDA, 2007) [38] and the Clinical and Laboratory Standards Institute (CLSI, 2008) [39]. The investigation of the CST was registered with the corresponding authorities (DIMDI: DE/CA37/IVD/7/8) and ethics commission as a medical product (MPG/in vitro diagnostic study). The STROBE statement checklist was strictly followed (Supplementary File S1).

### 4.3. Previous Systemic Periodontal Therapy

After first microbiological testing (T0), all patients (healthy subjects from former study were excluded; for in- and exclusion criteria of patients, see Arweiler et al. (2020) [5]) went through the standard pretreatment program (initial therapy I) of the department, with supragingival biofilm management, intensive instructions, and motivation for home-care biofilm management, including individual aids (such as interdental brushes) complemented by risk management (such as diabetes management or smoking cessation). 

After anti-infective treatment and re-evaluation, only a part of the subjects adhered to the supportive therapy (SPT) that was offered by the department, as described in the results. However, this was not an inclusion criterium to be included in this follow-up.

As to SRP, all participants had received the following therapy including subgingival debridement using Gracey curettes (Hu-Friedy, Chicago, IL, USA) and an ultrasonic device (Airflow Master Piezon, Electro Medical Systems, Nyon, Switzerland) under local anesthesia (if necessary) at sites with ≥4 mm probing pocket depth. Additional antibiotic therapy was administered according to clinical appearance and with regard to the microbiological result:

- SRP only (*n* = 44): when no *P. gingivalis* and no *A. actinomycetemcomitans* were detected, possible detection of *T. denticola*, *T. forsythia* and/or *P. intermedia*;
- SRP + M/A (*n* = 10): when *P. gingivalis* and *A. actinomycetemcomitans* and possibly *T. denticola*, *T. forsythia*, and/or *P. intermedia* were detected, administration of Amoxicillin 500 mg and Metronidazole 400 mg, each 3 times per day for 7 days.

→in case of penicillin allergy (*n* = 6): Clindamycin 600 mg, 3 times per day for 7 days [40].

- SRP + A (*n* = 11): When *P. gingivalis*, but no *A. actinomycetemcomitans* and possibly *T. denticola*, *T. forsythia*, and/or *P. intermedia*, was detected [34], administration of 500 mg, once per day (for 3 days) Azithromycin;
- SRP + local D (*n* = 3): when only 4–5 pockets were present and no *A. actinomycetemcomitans* was detected, 14% Doxycycline gel (Ligosan^®^ slow release, Heraeus Kulzer, Hanau, Germany) was locally administered [41].

### 4.4. Sample Collection for CST and qPCR

All patients enrolled were not allowed to use local antiseptics or antibacterial agents for 6 weeks or antibiotics for 6 months before assessment, since these could have influenced microbiological testing. 

The sample collection was performed by a dentist specialized in periodontology (AT). She was trained with different routine patients before the start of the study. In each patient, the same two sampling sites were specified as in the previous study, dried using cotton rolls, and carefully debrided supragingivally. A sterile paper point was then placed with a sterile tweezer into the base of the pocket for approximately 20 s in order to absorb sulcular fluid (subgingival plaque sample). In possibly healed healthy pockets, paper points were inserted 1–2 mm into the sulcus. Two paper points of each patient were pooled, filled in a reaction tube that was prefilled with glass-beads, and handed as previously described [5]. NBA and TMA performed the CST in this follow-up again, as previously in the initial study. Both were calibrated by the manufacturer. 

**Figure 3:**
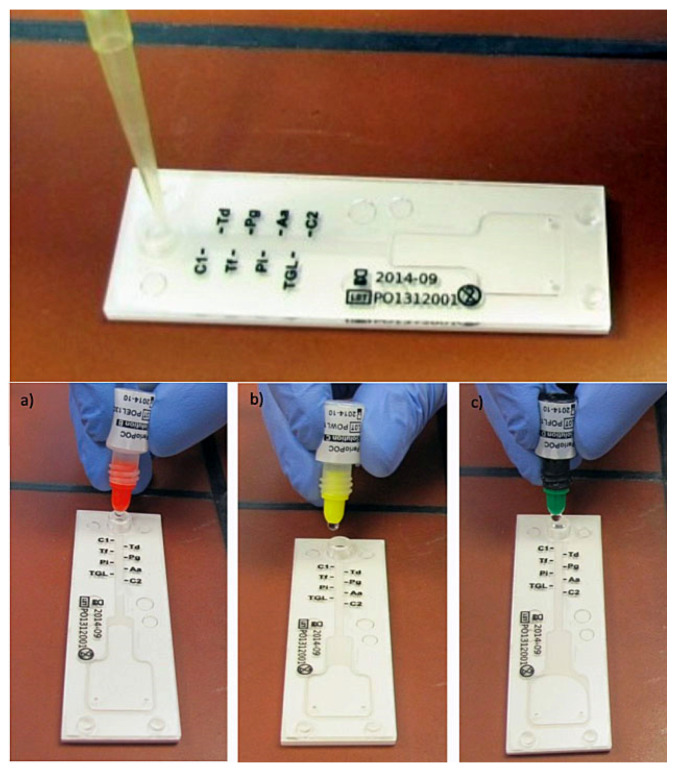
CST processing: application of the sample into the reaction tube of the test-kit, followed by reaction solutions: hybridization (**a**), washing (**b**), and staining (**c**).

In short, nucleic acids were denaturated with a lysis solution and heat-treated in boiling water. Hot lysate was then given into the inlet of the test chip. Hybridization, washing, and staining were performed by different solutions that were dropped into the reaction channel. After a further reaction time of 4 min, results on test chips could be read (Figure 3). A weak or strong blue coloration presented a positive reaction, meaning that the corresponding bacteria were present in a detection limit of about 10^4^ CFU/per probe. The leftover lysate (circa 120 µL each) was sealed tightly, frozen (−20 °C), and shipped on dry-ice to an external laboratory. Here, the quantitative real-time PCR (qPCR) as a reference method with a detection limit of 10^2^ CFU/probe was performed. 

### 4.5. Sample Size, Data Management and Data Collection Forms

A sample size calculation using simulations of probit analysis for estimation of limit of detection (LoD) was performed for the initial study (ACOMED Statistik, Leipzig, Germany) and revealed that ‘a width of the 95% confidence interval (CI) of about 0.5 units can be established, when a sample size of 50 is used’. Already, for the initial study, a (much larger) sample size of 100 periodontally diseased patients was selected from the pool of patients at the Department of Periodontology.

The present follow-up study with 74 patients still outnumbered the required sample size.

Only the groups, when subdivided by different therapies (e.g., local antibiotics), are very small, but the data are presented descriptively.

Data regarding patients’ periodontal treatments were documented pseudonymized on data collection sheets. CST and qPCR data were merged into Excel sheets (Microsoft Excel^®^, Redmond, WA, USA) and sent pseudonymized to ACOMED Statistik (Leipzig, Germany), who completed descriptive statistical data analysis.

### 4.6. Statistical Analysis

The primary variable was the elimination of bacteria (in %) for each single species with regard to therapy (before and after; T0/T1), determined by CST and qPCR as reference. The secondary parameter was the agreement of both test systems depending on the different therapy options.

## 5. Conclusions

Although neither conventional scaling and root planing nor the additional use of antibiotics (within correct indication) were able to eliminate five periodontal pathogens or prevent pocket reinfection, both test systems showed different elimination rates. This can be explained not only by different detection limits, but also by differences in detecting living and dead bacteria, which are highly amplified by qPCR and could lead to overestimation, especially after therapy. 

With regard to personalized medicine, microbiological tests can be not only a tool for first diagnostic but also to re-evaluate patients’ risk profiles after an anti-infective therapy. In this context, CSTs present an alternative with the advantage of immediate results. 

## Figures and Tables

**Table 1:** Overall elimination rate (in %) by CST and qPCR (at least positive for one of the five bacteria) resulting from samples positive at T1 in relation to T0 depending on different treatment modalities (SRP: scaling and root planing + A/M: Amoxicillin/Metronidazole, A: Azithromycin, C: Clindamycin, D: local Doxycycline) as well as agreement (in %) between both test systems.

Therapy	CST		qPCR		CST vs. qPCR
	Elimination%	T1/T0	Elimination%	T1/T0	Agreement%
SRP	59.1	18/44	29.6	31/44	75.5
SRP + A/M	90.0	1/10	30.0	7/10	82.2
SRP + A	72.8	3/11	9.1	10/11	65.5
SRP + C	66.7	2/6	33.3	4/6	90.0
SRP + D	66.7	1/3	33.3	2/3	40.0

**Table 2:** Elimination rate (in %) for single bacteria by CST and qPCR resulting from samples positive at T1 in relation to T0 depending from different treatment modalities (SRP: scaling and root planing + A/M: Amoxicillin/Metronidazole, A: Azithromycin, C: Clindamycin, D: local Doxycycline; *A.a.*: *Aggregatibacter actinomycetemcomitans*, *P.g.*: *Porphyromonas gingivalis*, *T.f.*: *Tannerella forsythia*, *T.d.*: *Treponema denticola*, *and P.i.*: *Prevotella intermedia*); n.a.: not applicable since bacterium was not detected at baseline.

	CST										qPCR									
	*A.a.*		*P.g.*		*P.i.*		*T.d.*		*T.f.*		*A.a.*		*P.g.*		*P.i.*		*T.d.*		*T.f.*	
	%	T1/T0	%	T1/T0	%	T1/T0	%	T1/T0	%	T1/T0	%	T1/T0	%	T1/T0	%	T1/T0	%	T1/T0	%	T1/T0
SRP	n.a	0/0	80.7	5/26	77.8	2/9	43.3	17/30	78.8	7/33	100	0/1	33.3	20/30	22.2	14/18	26.2	31/42	22.5	31/40
SRP + A/M	100	0/3	100	0/6	80.0	1/5	80.7	5/26	77.8	2/9	80.0	1/5	77.8	2/9	50.0	3/6	60.0	4/10	50.0	5/10
SRP + A	n.a	0/0	40.0	3/5	100	0/2	80.7	5/26	77.8	2/9	100	0/1	45.4	6/11	100	8/8	18.1	9/11	36.3	7/11
SRP + C	n.a	0/0	100	0/5	100	0/2	80.7	5/26	77.8	2/9	100	0/2	100	0/6	66.6	1/3	33.3	4/6	66.6	2/6
SRP + D	n.a	0/0	n.a	0/0	n.a	0/0	50.0	1/2	n.a	0/0	n.a	0/0	0	2/2	n.a	0/0	33.3	2/3	0	3/3

## Supplementary Materials

The following are available online at https://www.mdpi.com/article/10.3390/antibiotics11050577/s1, File S1: STROBE Statement—checklist of items that should be included in reports of observational studies.
